# GSTP1 knockdown induces metabolic changes affecting energy production and lipid balance in pancreatic cancer cells

**DOI:** 10.1080/23723556.2025.2518773

**Published:** 2025-06-14

**Authors:** Jenna N. Duttenhefner, Katie M. Reindl

**Affiliations:** Department of Biological Sciences, North Dakota State University, Fargo, ND, USA

**Keywords:** Pancreatic ductal adenocarcinoma, glutathione S-transferase pi 1 (GSTP1), metabolic reprogramming, metabolomics, therapeutic targeting

## Abstract

Pancreatic ductal adenocarcinoma (PDAC) is an aggressive cancer with limited treatment options, underscoring the need for novel therapeutic targets. Metabolic reprogramming is a hallmark of PDAC, enabling tumor cells to sustain rapid proliferation and survive under nutrient-deprived conditions. While glutathione S-transferase pi 1 (GSTP1) is a known regulator of redox homeostasis in PDAC, its role in metabolic adaptation remains unclear. Here, we show that GSTP1 knockdown disrupts PDAC metabolism, leading to downregulation of key metabolic enzymes (ALDH7A1, CPT1A, SLC2A3, PGM1), ATP depletion, mitochondrial dysfunction, and phospholipid remodeling. Phospholipid remodeling, including an increase in phosphatidylcholine (PC) levels, further suggests a compensatory response to metabolic stress. Importantly, GSTP1 knockdown led to elevated lipid peroxidation, increasing 4-hydroxynonenal (4-HNE) accumulation. Treatment with the antioxidant N-acetyl cysteine (NAC) partially restored metabolic gene expression, reinforcing GSTP1’s role in the interplay between redox regulation and metabolism in PDAC. By disrupting multiple metabolic pathways, GSTP1 depletion creates potential therapeutic vulnerabilities that could be targeted through metabolic and oxidative stress-inducing therapies to enhance treatment efficacy.

## 1. Introduction

Cancer cells must continuously adapt to hostile conditions, including nutrient deprivation, oxidative stress, and fluctuating energy demands. One way tumors overcome these challenges is through metabolic reprogramming, a hallmark of cancer that sustains proliferation and survival.^1^ In pancreatic ductal adenocarcinoma (PDAC), these metabolic adaptations are particularly pronounced, as tumors thrive in a dense, hypoxic microenvironment with limited nutrient availability.^2,3^ PDAC cells exhibit remarkable metabolic plasticity, allowing them to switch between different energy sources based on availability and demand. This adaptability is driven by dynamic mitochondrial metabolism, which plays a pivotal role in ATP production and biosynthetic precursor generation.^4,5^ Key pathways, including glycolysis, the tricarboxylic acid (TCA) cycle, and oxidative phosphorylation, are rewired to support PDAC’s aggressive nature and therapeutic resistance.

A defining feature of PDAC metabolism is the Warburg effect, where cancer cells preferentially rely on glycolysis for ATP production, even in the presence of oxygen.^6^ This shift supports biosynthetic needs by providing nucleotide, amino acid, and lipid synthesis intermediates. In parallel, the pentose phosphate pathway is upregulated, generating NADPH for antioxidant defense and macromolecule biosynthesis.^7^ However, PDAC metabolism extends beyond glycolysis, incorporating oxidative phosphorylation, the tricarboxylic acid cycle, and fatty acid oxidation to maintain metabolic flexibility.^8^ Mitochondria are central in coordinating these adaptations, integrating bioenergetic and redox signaling pathways.^9,10^ While oxidative phosphorylation remains functional in PDAC, its contribution varies among tumor subtypes, with some cells exhibiting high oxidative phosphorylation dependency.^11,12^ While these adaptations enhance PDAC cell survival, they also create metabolic vulnerabilities that could be therapeutically targeted.

PDAC has a dismal 5-year survival rate of just 13%.^13^ Effective screening options are not available, and most patients are diagnosed at late stages, with 25–30% presenting with locally advanced and 45–50% with metastatic disease.^14^ Despite significant advances in oncology, PDAC incidence and mortality rates continue to rise each year.^13^ Even among patients eligible for surgical resection, recurrence rates exceed 80% within two years, highlighting the urgent need for novel therapeutic targets.^15^ Although KRAS mutations, present in over 90% of PDAC cases, drive many metabolic alterations, recent evidence suggests additional regulators are involved.^7,16–18^ One such candidate is glutathione S-transferase pi 1 (GSTP1), a phase II detoxification enzyme best known for its role in protecting cells from oxidative stress.^19,20^ GSTP1 is frequently overexpressed in PDAC, where it has been linked to chemoresistance, tumor progression, and redox homeostasis.^20–22^ However, beyond its antioxidant function, GSTP1 may also play a critical role in metabolic regulation, a connection that remains largely unexplored in PDAC.^21,23^ Furthermore, analysis of gene expression and survival data from The Human Protein Atlas revealed that elevated GSTP1 expression is negatively correlated with PDAC patient survival post-diagnosis.^20^ These findings underscore the clinical relevance of GSTP1 in pancreatic cancer and provide a rationale for investigating its functional role in tumor metabolism.

Our previous multiomics analysis demonstrated the critical role of GSTP1 in PDAC cell survival, metabolic regulation, and redox homeostasis.^24^ GSTP1 knockdown induced significant oxidative stress, leading to compensatory metabolic adaptations, including upregulation of key regulators of oxidative stress response in PDAC. However, the precise metabolic mechanisms through which GSTP1 modulates PDAC metabolism remained unclear. Building on this evidence, the present study investigates the metabolic consequences of GSTP1 loss in PDAC cells, focusing on energy metabolism and lipid homeostasis. Using multiomic approaches, we demonstrate that GSTP1 knockdown disrupts glycolysis, mitochondrial function, and lipid metabolism, leading to ATP depletion and alterations in cellular lipid composition. These findings position GSTP1 as a central regulator of PDAC metabolic flexibility, linking redox balance with tumor bioenergetics. Given the increasing interest in targeting metabolic dependencies in PDAC, our study highlights that GSTP1 inhibition in combination with metabolic targeted therapies may offer a more effective approach to enhance therapeutic efficacy and overcome PDAC resistance mechanisms.

## 2. Materials and methods

### 2.1. Reagents

Puromycin (MSPPANTPR1) was acquired from Sigma-Aldrich. Doxycycline (198955) was purchased from MP Biomedicals. Antibodies against ALDH7A1 (Antiquitin, sc -514,167), GSTP1 (sc -376,013), SLC2A3 (GLUT3, sc -74,497), and PGM1 (sc -373,796) were sourced from Santa Cruz Biotechnology, while 4-hydroxynonenal (4-HNE, ab46545) was purchased from Abcam. Antibodies targeting CPT1A (12252S), GAPDH (97166S), β-actin (4970S), and horseradish peroxidase (HRP)-conjugated secondary antibodies (Rabbit-7074S, Mouse-7076S, Biotin-7075P5) were obtained from Cell Signaling Technology. N-acetyl cysteine (NAC, A916S) was purchased from Sigma Aldrich.

### 2.2. Cell culture

PDAC cell lines (MIA PaCa-2, PANC-1, and HPAF-II) were obtained from the American Type Culture Collection (ATCC). MIA PaCa-2 cells were cultured in Dulbecco’s Modified Eagle Medium (DMEM) characterized by high glucose content (Corning, 10–013-CV), supplemented with 10% (v/v) fetal bovine serum (FBS) (Atlanta Biologicals, S11150H) and 2.5% (v/v) horse serum (Corning, SH30074.03). PANC-1 and HPAF-II cells were maintained in DMEM high-glucose and Eagle’s Minimum Essential Medium (EMEM) (Corning, 10–009-CV) media, respectively, supplemented with 10% (v/v) FBS. All cultures were incubated at 37 °C with 5% CO_2_ and contained 1% HyClone Antibiotic Antimycotic (Penicillin/Streptomycin/Fungizone) Solution (Corning, 30–004-CI). Upon reaching 80% confluency, the cell lines were subcultured through enzymatic digestion with 0.25% trypsin/1 mm EDTA solution (Corning, 25–051-CI). Lentiviral-transfected cells (nonspecific (NS) scramble shRNA control and GSTP1 knockdown (shGSTP1–1)) were produced as described^24^ and were cultured in growth media containing 5 µg/mL puromycin to maintain selection pressure in transfected cells.

### 2.3. Protein extraction and western blotting

Cells were washed with cold PBS (Corning, 21–040-CV) and lysed using cell culture lysis buffer (Invitrogen, FNN0011) supplemented with a protease/phosphatase inhibitor cocktail (Cell Signaling Technology, 5872S). Lysates were incubated on ice for 30 minutes, centrifuged (13,000 rpm, 10 minutes, 4°C), and protein supernatants were collected. Protein concentrations were determined using the Pierce^TM^ BCA Protein Assay Kit (Thermo Fisher Scientific 23,225). Protein samples (10–80 µg) were prepared in Laemmli SDS sample buffer (Thermo Fisher Scientific, J60015.AC) with 3–5% BME, denatured at 100°C, resolved on 7–10% SDS-polyacrylamide gels (100 V, 2.5–3 hours, 4°C), and transferred to a nitrocellulose membrane (GE Healthcare Life Sciences 10,600,002) (100 V, 70 minutes, 4°C). Blots were blocked (5% BSA, 3 hours), incubated overnight (4°C) with a primary antibody (1:1000), washed with TBS-T, and probed with HRP-inked secondary antibodies containing anti-biotin (1:2000, 1:5000) for 1 hour at room temperature. Blots were developed using SuperSignal West Femto Maximum Sensitivity Substrate (Thermo Fisher Scientific 34,096) and imaged on a FluorChem® FC2 Imaging System (Alpha Innotech). Densitometry analysis for quantification was performed using ImageJ software.

### 2.4. Quantitative real-time PCR

RNA was extracted using the Phenol-Free Total RNA Purification Kit (VWR Life Science 121,830,194) per the manufacturer’s instructions and was eluted in 50 µL of nuclease-free water. The RNA concentrations were measured using a NanoDrop 1000 Spectrophotometer (Thermo Fisher Scientific), and 2 µg of RNA was converted to cDNA using the qScript cDNA synthesis kit (Quanta Biosciences 95,047–100). Real-time qPCR was performed in triplicate using PerfeCTa® SYBR® Green Supermix (Quanta Biosciences 95,053), a 1:10 dilution of cDNA, and 3 mm forward and reverse primers (Table 1). Primers were constructed using the PrimerQuest^TM^ tool and synthesized by Integrated DNA Technologies. The 96-well PCR microplate (Sigma Aldrich) was processed using the Stratagene Mx3000P® Multiplex Quantitative PCR System (Agilent Technologies) under the following thermal cycling conditions: 95°C (2 minutes), 45 cycles of 95°C (15 seconds), 55°C (30 seconds), and 72°C (30 seconds). Data were normalized utilizing β-actin and β-tubulin as housekeeping reference genes, with data analysis performed via the 2-ΔΔCt method.^25^

**Table 1.** Primer sequences used for measuring mRNA expression via quantitative polymerase chain reaction.

Gene	Forward primer	Reverse primer
GSTP1	5’-CAG GAG GGC TCA CTC AAA GC-3′	5’-AGG TGA CGC AGG ATG GTA TTG-3′
ALDH7A1	5’-TTT CCC TGT GGC AGT GTA TG-3’	5’-CCT CCA GAA CCT TGG CTA TTA TC-3’
CPT1A	5’-TCC TGG TGG GCT ACA AAT TAC-3’	5’-CCT GAA TGT GAG TTG GAA GGA −3’
SLC2A3	5’-TAC CAT CCT TCC TGC TAT CCT-3’	5’-GAC ATC CTT GCA CTC TCA TCT *T*-3’
PGM1	5’-GTT GAC GAG CAG TGC ATT TAC-3’	5’-CCT GAT GGC TAA GGA GAC AAA TA-3’
β-Actin	5’-TTG CCG ACA GGA TGC AGA A-3′	5’-GCC GAT CCA CAC GGA GTA CTT-3′
β-Tubulin	5’-GTT CGC TCA GGT CCT TTT GG-3′	5’-CCC TCT GTG TAG TGG CCT TTG-3′

### 2.5. Comprehensive hydrophilic metabolites panel

#### 2.5.1. Sample preparation and LC-MS analysis

Following GSTP1 knockdown for 96 hours, hydrophilic metabolites were extracted from five replicates of NS control and shGSTP1–1 MIA PaCa-2 cells following Northwestern University Metabolomics Core Facility protocols. Lysates were stored at −80°C, with pellets reserved for protein quantification. The protein pellet was fully dissolved in 8 M urea buffer, and protein quantitation was completed using the Pierce™ BCA Protein Assay Kit (Thermo Fisher Scientific) according to the manufacturer’s instructions. The metabolite-containing supernatant was transferred on dry ice to the Metabolomics Core Facility (MCF) at Robert H. Lurie Comprehensive Cancer Center of Northwestern University, Chicago, IL, with corresponding protein quantitation data. LC-MS detected over 150 water-soluble metabolites, normalized using the total ion count (TIC)/lowest TIC in the batch.

#### 2.5.2. Differential metabolite level analysis

MetaboAnalyst was used for comprehensive metabolomic data analysis. Metabolite MS peak area values were assessed for quality using MetaboAnalyst integrated data integrity check. Quality control was conducted using interquartile range (IQR) filtering to remove low-repeatability variables. Principle component analysis (PCA) and heatmap analysis identified one NS control sample as an outlier, resulting in removal from downstream analysis. Data were normalized using the provided normalization factor for each sample (TIC/lowest TIC in batch). Metabolites considered significantly different between NS control and shGSTP1–1 knockdown groups were identified (p-value <.05, fold change > 2). Functional pathway analysis was performed using MetaboAnalyst tools.

### 2.6. ATP quantification assay- CellTiter-Glo® assay

MIA PaCa-2, PANC-1, and HPAF-II PDAC cell lines, with either control (NS) or GSTP1 knockdown (shGSTP1–1), were seeded into 96-well plates. After 24 hours, doxycycline (500 ng/mL) was added, and cells were cultured for 96 hours until reaching 70–80% confluence. Before the assay, cells and CellTiter-Glo reagent were equilibrated to room temperature. Hoechst dye was added to all wells to a final concentration of 1 μg/mL and incubated for 1 hour at 37°C. The media was removed, cells were washed with PBS, and fresh DMEM (no phenol red + hEPES) was added (Agilent Technologies 103,575–100). Hoechst 33,342 (Cayman Chemicals 15,547) fluorescence (Ex/Em = 350/461 nm) was measured for cell number normalization using the BioTek Cytation 5 Cell Imaging Multimode Reader (Agilent Technologies). An equal volume of CellTiter-Glo reagent (Promega, G7572) was added to each well and mixed at 500–700 rpm for 2 minutes to facilitate cell lysis. The cells were then incubated at room temperature for 10 minutes to stabilize the luminescent signal before measuring luminescence using the BioTek Cytation 5 Cell Imaging Multimode Reader (Agilent Technologies).

### 2.7. Mitochondrial membrane potential (δψm) – TMRE assay

MIA PaCa-2, PANC-1, and HPAF-II NS and shGSTP1–1 cells were seeded in 96-well plates with three technical replicates per treatment. Control treatments included positive, carbonyl cyanide 4-trifluoromethoxy phenylhydrazone (FCCP) (VWR 76,800–132) and negative, no tetramethyl rhodamine ethyl ester (TMRE) controls. After 24 hours, all cells were treated with 500 ng/mL doxycycline to induce GSTP1 knockdown, and cells were incubated for 96 hours to near 90–95% confluency. All subsequent steps were completed in the dark. Positive control wells were treated with FCCP to a final concentration of 20 μM and incubated for 10 minutes at 37°C. Subsequently, media was removed from all wells and replaced with corresponding 400 μM TMRE (AAT ioQuest 22,220) + 5 μg/mL u media or no TMRE +5 μg/mL Hoechst media. After 30 minutes of incubation at 37°C, media was aspirated, wells were washed twice with PBS, and plates were equilibrated to room temperature for 15–30 minutes. The fluorescence was measured at Ex/Em = 549/575 nm for TMRE and Ex/Em = 350/461 nm for Hoechst cell number normalization using the BioTek Cytation 5 Cell Imaging Multimode Reader (Agilent Technologies). The data represent the average ± standard deviation of three independent experiments for the three PDAC cell lines.

### 2.8. Comprehensive phospholipid panel

#### 2.8.1. Sample preparation and LC-MS analysis

MIA PaCa-2 NS control and shGSTP1–1 cells were treated with doxycycline for 96 hours. Cell counts were obtained, and pellets were snap-frozen and stored at −80°C until transfer to the University of California San Diego Lipidomics Core, La Jolla, CA, for extraction and LC-MS analysis.^26^ Five biological replicates from NS-control and sh-GSTP1–1 knockdown groups were submitted for analysis. Samples were extracted at the facility using the BUME method. The lipid fraction was reconstituted in 18:1:1 IPA/DCM/MeOH. Liquid Chromatography analysis was performed on RP-UPLC/MS Thermo Vanquish UPLC (ThermoFisher Scientific). Mass spectrometry was completed using MS/MS data-dependent acquisition on the Thermo Q Exactive Mass Spectrometer (ThermoFisher Scientific, Waltham). In-house analysis was run using the Lipid Data Analyzer (LDA) and Lipid Search software packages. Metabolite coverage percentage for each class of target phospholipid was confirmed using MS/MS footprinting fragmentation.

#### 2.8.2. Class profile analysis

All data were presented as normalized intensities relative to the measured internal standards, and the relative abundances of target phospholipid per mg of protein were represented. Semi-quantitative results allow for direct comparison of individual metabolites between samples and different metabolites in the same sample. Phospholipid classes were resolved at the level of fatty acid composition and include phosphatidylcholine (PC), plasmenyl phosphatidylcholine (P-PC), lyso-phosphatidylcholine (LPC), phosphatidylethanolamine (PE), plasmenyl phosphatidylethanolamine (P-PE), lyso-phosphatidylethanolamine (LPE), phosphatidylserine (PS), lyso-phosphatidylserine (LPS), phosphatidylinositol (PI), and phosphatidylglycerol (PG). Metabolite classes with an FDR-adjusted p-value of less than or equal to 0.01 and a fold change greater than two were identified as significantly differentially expressed between the NS control and shGSTP1–1 knockdown cells.

### 2.9. Statistical analysis

Statistical analyses were performed in GraphPad Prism 10. The results are based on at least three biological experiments, each with at least three technical replicates, and are shown as the mean ± standard deviation. A Student’s t-test was used to compare the control (NS) and knockdown (shGSTP1) groups, with *p* < .05 considered significant, except for the LC-MS comprehensive phospholipid panel, where an FDR-adjusted *p* < .01 was used. Cell proliferation was analyzed for each PDAC cell line, considering the knockdown, time, and replicates as factors.

## 3. Results

### 3.1. GSTP1 knockdown disrupts core metabolic pathways in PDAC cells

**Figure 1.**
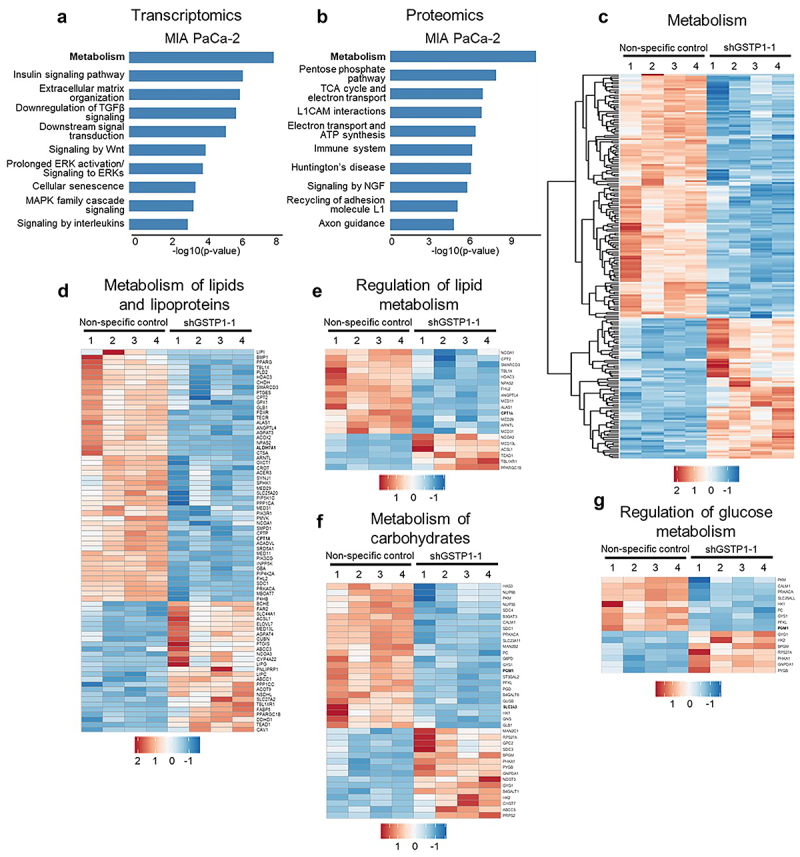
GSTP1 knockdown disrupts core metabolic pathways in PDAC cells. (a-b) comparative transcriptomic and proteomic analysis identified metabolism as the most dysregulated pathway following GSTP1 knockdown in MIA PaCa-2 cells (*n* = 4). (c) Pathway enrichment analysis reveals significant alterations in cellular metabolism, including glycolysis, oxidative phosphorylation, the pentose phosphate pathway, and the TCA cycle. (d) Lipid metabolism is significantly disrupted, with downregulation of genes associated with lipid and lipoprotein metabolism and (e) regulation of lipid metabolism, (f) carbohydrate metabolism is also affected with dysregulated expression of (f) key regulators of glucose metabolism identified through RNA-Seq. Blue indicates downregulated genes in GSTP1 knockdown (shGSTP1–1) MIA PaCa-2 cells compared to the nonspecific control, while red indicates upregulated genes.

To investigate the role of GSTP1 in PDAC metabolism, we employed a doxycycline-inducible knockdown system in PDAC cell lines (MIA PaCa-2, PAC-1, HPAF-II), as previously described.^24^ Doxycycline treatment (500 ng/mL) was applied to both GSTP1-targeting and control shRNA cell lines. Prior optimization confirmed that this dose had no effect on GSTP1 expression or cellular viability in control cells, as previously demonstrated by our group,^24^ and was used consistently across all experiments. Upon doxycycline treatment, GSTP1 expression was significantly reduced, allowing us to assess its impact on metabolic pathways. Building on our previous multiomics study,^24^ which established GSTP1 as a key regulator of oxidative stress and metabolic adaptation in PDAC, we performed pathway enrichment analysis to further explore the metabolic consequences of GSTP1 depletion. Comparative transcriptomic and proteomic analysis revealed that metabolism was the most dysregulated pathway following GSTP1 knockdown (Figure 1a,b). Specifically, genes associated with cellular metabolism, oxidative phosphorylation, ATP synthesis, the pentose phosphate pathway, and the TCA cycle were significantly altered (Figure 1c). Additionally, GSTP1 knockdown disrupted lipid metabolism and regulation in GSTP1 knockdown cells (Figure 1d,e), affecting pathways involved in lipid transport, lipid digestion, and metabolism of glycerophospholipids, phospholipids, fatty acids, triglycerides, and ketone bodies (Figure S1A-B). In parallel, genes associated with carbohydrate metabolism, including glycolysis, glucose transport, and the TCA cycle, were significantly dysregulated (Figure 1f,g, S1C-E). These findings suggest that GSTP1 is a critical metabolic regulator in PDAC cells, maintaining energy homeostasis by coordinating lipid and carbohydrate metabolism.

### 3.2. GSTP1 knockdown leads to downregulation of key metabolic regulators

**Figure 2.**
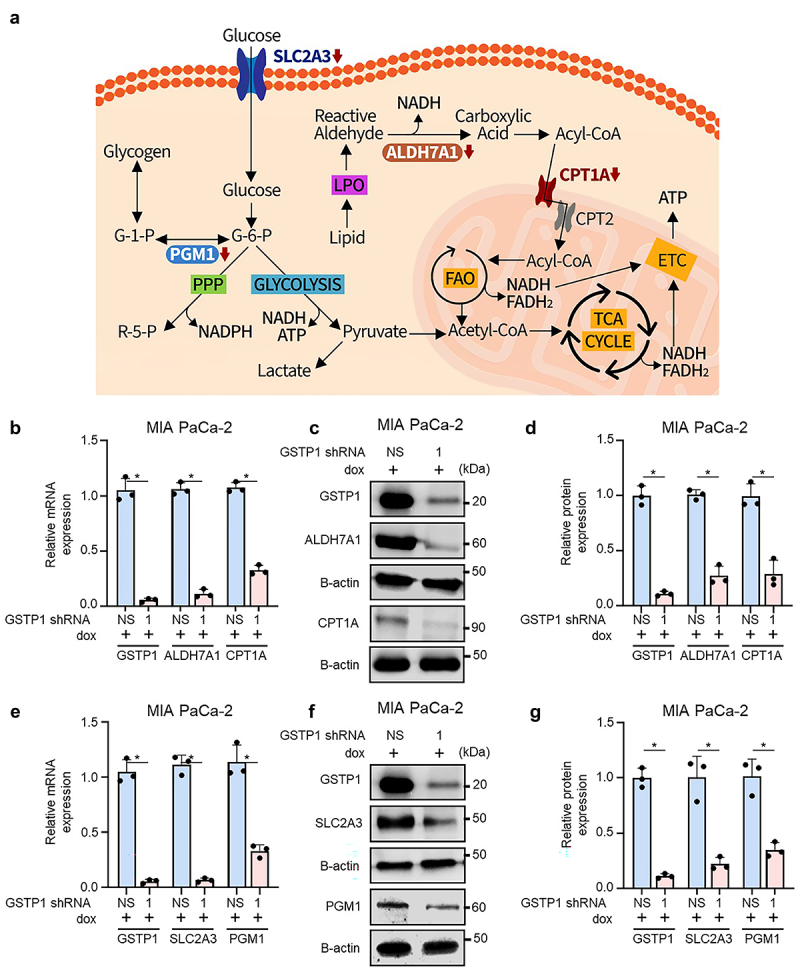
GSTP1 knockdown reduces the expression of key metabolic regulators. (a) Schematic representation of ALDH7A1, CPT1A, SLC2A3, and PGM1, highlighting their roles in lipid and carbohydrate metabolism. (b) qRT-PCR expression analysis shows significant reductions in ALDH7A1 and CPT1A mRNA expression in MIA PaCa-2 cells following GSTP1 knockdown (shRNA-1) compared to the nonspecific control (shRNA-NS). (c-d) western blot validation confirms decreased ALDH7A1 and CPT1A protein levels. (e) SLC2A3 and PGM1 mRNA expression is significantly downregulated upon GSTP1 loss. (f-g) western blot analysis confirms the downregulation of SLC2A3 and PGM1 proteins. The figures shown are indicative of three separate experiments (*n* = 3). To assess the statistical significance of expression differences between groups, the Student’s t-test was employed. Statistically significant variations between GSTP1 knockdown and the control group are indicated by an asterisk (*), with a p-value less than 0.05.

To further characterize the metabolic consequences of GSTP1 loss, we examined the expression of key metabolic genes and proteins downregulated by GSTP1 knockdown, focusing on aldehyde dehydrogenase 7 family member A1 (ALDH7A1) and carnitine palmitoyltransferase 1A (CPT1A) due to their roles in lipid metabolism and solute carrier family 2 member 3 (SLC2A3) and phosphoglucomutase 1 (PGM1) for their involvement in glucose metabolism. A schematic of their roles in lipid and glucose metabolism is shown in Figure 2a. Following GSTP1 depletion, significant reductions were observed in ALDH7A1, a mitochondrial enzyme involved in detoxifying lipid peroxidation, and CPT1A, which regulates mitochondrial fatty acid oxidation. Additionally, the expression of SLC2A3, a glucose transporter, and PGM1, which plays a role in glycogen metabolism and glycolysis, was decreased. These changes were confirmed in GSTP1 knockdown PDAC cells through qPCR and western blot analysis (see below).

#### 3.2.1. Lipid metabolism

In MIA PaCa-2 cells, ALDH7A1 mRNA and protein levels were reduced by 88% and 72%, respectively. CPT1A expression decreased by 72% at both the mRNA and protein levels (Figure 2b–d). Similar reductions were observed in PANC-1 and HPAF-II cells. In PANC-1 cells, ALDH7A1 mRNA and protein levels were reduced by 71%, while CPT1A mRNA decreased by 43% and protein levels by 62% (Figure S2A-C). In HPAF-II cells, ALDH7A1 mRNA and protein levels decreased by 73% and 72%, respectively, and CPT1A expression was reduced by 67% at the mRNA level and 69% at the protein level (Figure S2G-I).

#### 3.2.2. Carbohydrate metabolism

GSTP1 knockdown also significantly impaired glucose metabolism. In MIA PaCa-2 cells, SLC2A3 mRNA and protein levels were reduced by 93% and 81%, respectively, while PGM1 expression dropped by 66% at both mRNA and protein levels (Figure 2e–g). In PANC-1 cells, SLC2A3 mRNA and protein levels decreased by 70% and 47%, respectively, while PGM1 mRNA and protein levels were reduced by 52% and 57% (Figure S2J-L). These results indicate that GSTP1 knockdown significantly disrupts lipid and glucose metabolism, highlighting its essential role in maintaining metabolic homeostasis in PDAC cells.

### 3.3. GSTP1 recovery restores metabolic gene expression

To confirm the dependence of these metabolic changes on GSTP1, we performed a doxycycline recovery experiment. After 96 hours of GSTP1 knockdown, doxycycline was removed for 120 hours, allowing GSTP1 expression to recover. We then assessed the recovery of ALDH7A1, CPT1A, SLC2A3, and PGM1 at both the mRNA and protein levels.

#### 3.3.1. Recovery of lipid metabolism genes

**Figure 3.**
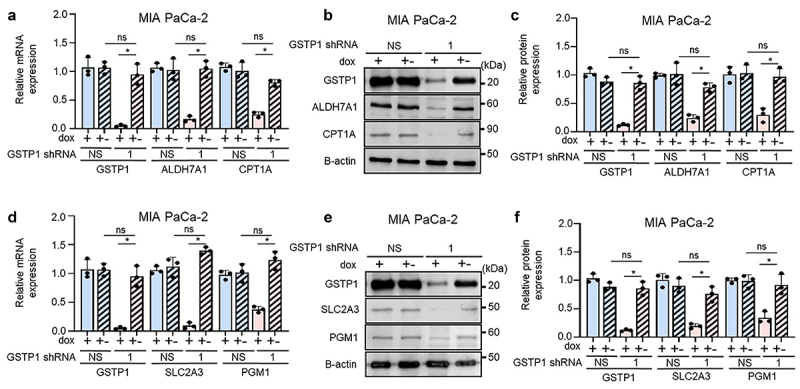
GSTP1 restoration reverses metabolic dysregulation in PDAC cells. (a) qRT-PCR demonstrates the recovery of ALDH7A1 and CPT1A mRNA levels following GSTP1 re-expression (dox ±). (b-c) western blot analysis confirms partial restoration of ALDH7A1 and CPT1A protein levels after GSTP1 restoration. (d) SLC2A3 and PGM1 mRNA levels are significantly recovered following GSTP1 restoration. (e-f) western blot confirmation of SLC2A3 and PGM1 protein recovery to varying degrees. Expression levels were compared between 120 hour NS recovery (NS ±) and 120 hour shGSTP1–1 recovery (shGSTP1–1 ±) as well as between shGSTP1–1 + and shGSTP1–1 recovery (shGSTP1 ±). Representative results from three independent experiments in MIA PaCa-2 cells are shown (*n* = 3). Statistical analysis was determined using the student’s t-test, with an asterisk (*) indicating significant differences (*p* < .05) between GSTP1 knockdown and control conditions.

Following GSTP1 restoration, ALDH7A1 and CPT1A expression are significantly recovered. In MIA PaCa-2 cells, ALDH7A1 mRNA expression fully recovered, slightly exceeding baseline levels (~102%), while protein levels were restored to 72%. CPT1A mRNA levels recovered to 65%, with protein levels nearly returning to baseline at 94% (Figure 3a–c). In PANC-1 cells, ALDH7A1 mRNA expression exceeded baseline levels at 132%, with protein expression levels reaching 96%. CPT1A mRNA expression recovered to 112%, with protein restoration at 96% (Figure S3A-C). In HPAF-II cells, ALDH7A1 mRNA expression returned to 121%, while protein levels recovered to 75% (Figure S3G-I). CPT1A mRNA levels recovered to 97%, while protein levels reached 70% (Figure S3G-I).

#### 3.3.2. Recovery of carbohydrate metabolism genes

SLC2A3 and PGM1 also showed substantial recovery following GSTP1 re-expression. In MIA PaCa-2 cells, SLC2A3 mRNA expression strongly rebounded to 140%, with protein levels recovering to 70% (Figure 3d–f). PGM1 mRNA expression recovered to 136%, while protein levels were restored to 89% (Figure 3d–f). In PANC-1 cells, SLC2A3 mRNA expression recovered to 85%, while protein levels reached 62% (Figure S3D-F). PGM1 mRNA levels were restored to 95%, with protein recovery at 73% (Figure S3D-F). In HPAF-II cells, SLC2A3 mRNA expression recovered to 85%, while protein levels exceeded baseline at 117% (Figure S3J-L). PGM1 mRNA levels were restored to 92%, with protein recovery reaching 103% (Figure S3J-L).

These results confirm that GSTP1 directly regulates key metabolic genes and that metabolic dysfunction following inducible GSTP1 knockdown is reversible upon re-expression. Interestingly, mRNA levels exceeded baseline, suggesting compensatory transcriptional upregulation, whereas protein recovery was more variable, suggesting post-transcriptional regulation.

### 3.4. GSTP1 knockdown increases lipid peroxidation and 4-HNE accumulation

**Figure 4.**
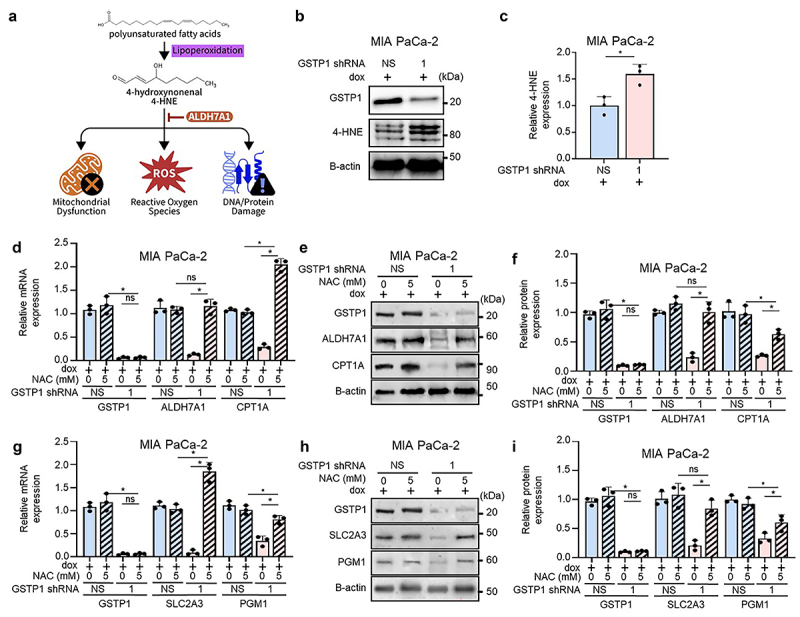
GSTP1 knockdown increases 4-HNE accumulation while antioxidant treatment partially restores metabolic gene expression. (a) Schematic representation of 4-HNE accumulation and its detrimental effects on mitochondrial function, ROS generation, and cellular damage. (b-c) western blot analysis shows a significant increase in 4-HNE expression following GSTP1 knockdown in MIA PaCa-2 cells, indicating elevated lipid peroxidation. (d) N-acetyl cysteine (NAC) treatment (48 hours, 5 mm) restores ALDH7A1 and CPT1A mRNA expression. (e-f) western blot validation of NAC-mediated partial recovery of ALDH7A1 and CPT1A protein levels. (g) SLC2A3 mRNA is restored with NAC treatment, though PGM1 recovery is less pronounced at both the mRNA and protein levels. (h-i) western blot analysis assessing NAC’s partial recovery effects on SLC2A3 and PGM1 protein levels. Expression levels were compared between NAC-treated NS control (NS 5 mm) and GSTP1–1 knockdown NAC-treated (shGSTP1–1 5 mm) as well as between untreated (0 mm) shGSTP1–1 and NAC-treated (5 mm) shGSTP1–1 conditions. Representative results from three independent experiments are shown (*n* = 3). Statistical analysis was determined using the student’s t-test, with an asterisk (*) indicating *p* < .05 between GSTP1 knockdown and control conditions.

Given the role of ALDH7A1 in lipid peroxidation detoxification, we next examined the effect of GSTP1 depletion of lipid peroxidation, focusing on 4-hydroxynonenal (4-HNE), a substrate of ALDH7A1. Accumulation of 4-HNE contributes to oxidative stress, mitochondrial dysfunction, ROS generation, and DNA/protein damage, ultimately disrupting cellular homeostasis. ALDH7A1 mitigates these effects by metabolizing 4-HNE, thereby reducing oxidative damage, supporting lipid metabolism, and preserving cellular function (Figure 4a). Our data show that GSTP1 knockdown significantly increases 4-HNE expression across all PDAC cell lines evaluated. MIA PaCa-2 cells exhibited a 1.6-fold increase (Figure 4b,c), while PANC-1 and HPAF-II showed 1.5-fold and 1.3-fold increases, respectively (Figure S4A-D). These findings indicate that GSTP1 is crucial for limiting lipid peroxidation and oxidative stress in PDAC cells, thereby preserving cellular function.

### 3.5. Antioxidant treatment partially restores GSTP1-dependent metabolic gene expression

Since GSTP1 inactivation is known to drive oxidative stress through both genetic^20,24^ and pharmacological^27,28^ mechanisms, we next investigated whether oxidative stress contributes to the metabolic changes observed upon GSTP1 depletion. We treated PDAC cells with N-acetyl cysteine (NAC), an antioxidant, for 48 hours. NAC functions both as a direct ROS scavenger and a reducing agent, breaking disulfide bonds to restore redox balance. More importantly, it serves as a precursor for L-cysteine, the rate-limiting substrate in glutathione synthesis.^29,30^ As the most abundant endogenous antioxidant, glutathione also plays a central role in neutralizing ROS and facilitating detoxification through glutathione S-transferases and glutathione peroxidases.^31^

#### 3.5.1. Recovery of lipid metabolism genes following NAC treatment

In MIA PaCa-2 cells, NAC treatment fully restored ALDH7A1 expression, with mRNA returning to 114% and protein levels returning to 101% (Figure 4d–f). CPT1A mRNA increased to 234%, although protein recovery was lower at 49% (Figure 4d–f). PANC-1 cells showed a 160% recovery of ALDH7A1 mRNA, while protein levels were restored to 81% (Figure S4E-G). CPT1A mRNA improved to 40% recovery, with protein levels reaching 25% (Figure S4E-G). In HPAF-II cells, ALDH7A1 mRNA expression rebounded to 112%, while protein levels were recovered to 62% (Figure S4K-M). CPT1A mRNA increased to 139%, with protein restoration at 64% (Figure S4K-M).

#### 3.5.2. Recovery of carbohydrate metabolism genes following NAC treatment

SLC2A3 and PGM1 also showed varying degrees of recovery, indicating that oxidative stress contributes to their regulation. MIA PaCa-2 cells exhibited a 183% recovery of SLC2A3 mRNA, while protein levels returned to 79% (Figure 4g–i). PGM1 recovery, however, was more moderate, with mRNA reaching 60% and protein increasing to 41% (Figure 4g–i). In PANC-1 cells, SLC2A3 mRNA increased to 182% recovery, with protein levels recovering to 79% (Figure S4H-J). Again, PGM1 exhibited a more moderate recovery of 44% at the mRNA level and 48% at the protein level (Figure S4H-J). HPAF-II cells showed the most pronounced SLC2A3 recovery, with mRNA exceeding baseline at 213% and protein levels reaching 125% (Figure S4N-P). However, PGM1 failed to recover at the mRNA level and showed only partial protein restoration at 11% (Figure S4N-P). These findings reinforce the role of GSTP1 in regulating oxidative stress and metabolic stability in PDAC cells with antioxidant treatment providing partial restoration of gene expression. However, the varying degrees of recovery suggest that additional regulatory mechanisms beyond oxidative stress may be involved in GSTP1-mediated metabolic control.

### 3.6. Metabolomic analysis reveals GSTP1 knockdown reduces ATP levels and disrupts mitochondrial function

**Figure 5.**
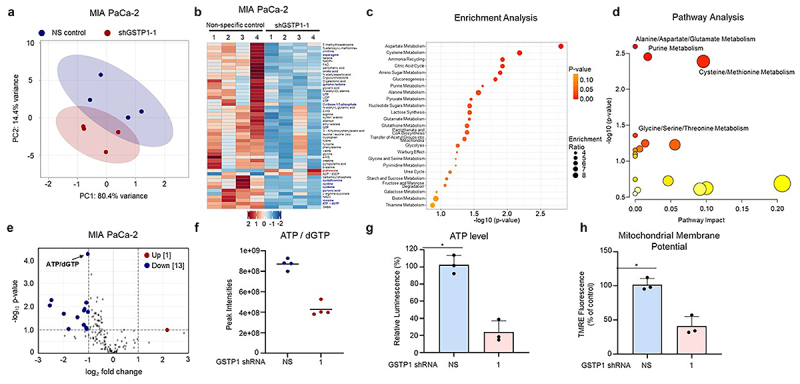
GSTP1 knockdown reduces ATP levels and disrupts mitochondrial function. (a) Principal component analysis (PCA) of metabolic profiles reveals distinct metabolic shifts between GSTP1 knockdown and control MIA PaCa-2 cells. (b) Heatmap of the top 50 differentially expressed metabolites highlights widespread metabolic suppression following GSTP1 knockdown. Blue color represents downregulation, while red color represents upregulation between NS control and GSTP1 knockdown cells (shGSTP1–1) (c) metabolite set enrichment analysis using the small molecule pathway database (SMPDB) shows the top 25 enriched pathways. (d) KEGG-based metabolic pathway analysis incorporated both enrichment and topology (impact) scores; pathways with *p* < .05 are labeled. In both plots, circle size indicates pathway impact and color intensity reflects statistical significance. Analyses were performed in MetaboAnalyst 6.0. (e-f) ATP/dGTP levels are significantly reduced (2.0-fold) in GSTP1 knockdown cells as determined by LC-MS and (g) a 4.3-fold decrease in ATP confirmed by CellTiter-Glo luminescence assays. (h) TMRE staining reveals a significant reduction in mitochondrial membrane potential, confirming mitochondrial dysfunction. Data are presented as representative results from three independent experiments (*n* = 3). Statistical significance was assessed using the student’s t-test, with * denoting a significant difference (*p* < .05) between GSTP1 knockdown and control conditions.

To investigate the metabolic consequences of GSTP1 knockdown, we conducted a comprehensive hydrophilic metabolite panel analysis on MIA PaCa-2 cells using liquid chromatography-mass spectrometry (LC-MS). This approach profiled over 250 water-soluble metabolites, including amino acids, nucleotides, cofactors, and the intermediates of central metabolic pathways such as the TCA cycle, glycolysis, gluconeogenesis, and the pentose phosphate pathway. Principle component analysis (PCA) revealed distinct metabolic shifts between GSTP1 knockdown and control cells, suggesting widespread metabolic alterations (Figure 5a). A heatmap of the top 50 differentially expressed metabolites showed a predominant downregulation of metabolites in GSTP1 knockdown cells, suggesting metabolic suppression due to GSTP1 loss (Figure 5b).

To further investigate the functional implications of metabolite changes following GSTP1 knockdown, we conducted pathway-level analysis using MetaboAnalyst. Metabolite Set Enrichment Analysis (MSEA) based on the Small Molecule Pathway Database (SMPDB) identified the top 25 enriched pathways, including those related to amino acid metabolism, energy production, and redox balance (Figure 5c). Complementary analysis using the Kyoto Encyclopedia of Genes and Genomes (KEGG) database integrated pathway enrichment with topological impact scoring, highlighting several significantly altered pathways with high biological relevance. Notably, pathways involved in amino acid metabolism and purine metabolism were significantly disrupted (Figure 5d). These pathways are essential for energy production, biosynthesis, and antioxidant defense, suggesting that GSTP1 knockdown disrupts metabolic homeostasis at a systems level.

Among the 14 significantly differentially expressed metabolites (*p* < .05, log_2_ fold change > ±1), ATP/dGTP exhibited the most pronounced reductions, showing a 2-fold decrease in GSTP1 knockdown cells (Figures 5e,f). This depletion was further validated by a CellTiter-Glo luminescence assay, which revealed a 4.3-fold decrease in ATP in MIA PaCa-2 cells (Figure 5g). Similarly, PANC-1 and HPAF-II cells showed 1.7-fold and 4.3-fold reductions, respectively (Figure S5A).

Given the essential role of mitochondrial function in sustaining ATP production, we next assessed mitochondrial integrity using TMRE assays. GSTP1 knockdown significantly reduced mitochondrial membrane potential across PDAC cell lines, with MIA PaCa-2 and HPAF-II cells exhibiting 60% and 45% reductions, respectively (Figures 5h, S5B). PANC-1 cells exhibited a moderate but significant 24.5% reduction (Figure S5B). The magnitude of mitochondrial dysregulation varied by cell line, suggesting cell-type-specific dependencies on GSTP1 for mitochondrial homeostasis. These findings demonstrate that GSTP1 knockdown leads to reduced mitochondrial membrane potential and ATP depletion in PDAC cells, indicating impaired mitochondrial function.

### 3.7. GSTP1 knockdown alters phospholipid metabolism and lipid homeostasis

**Figure 6.**
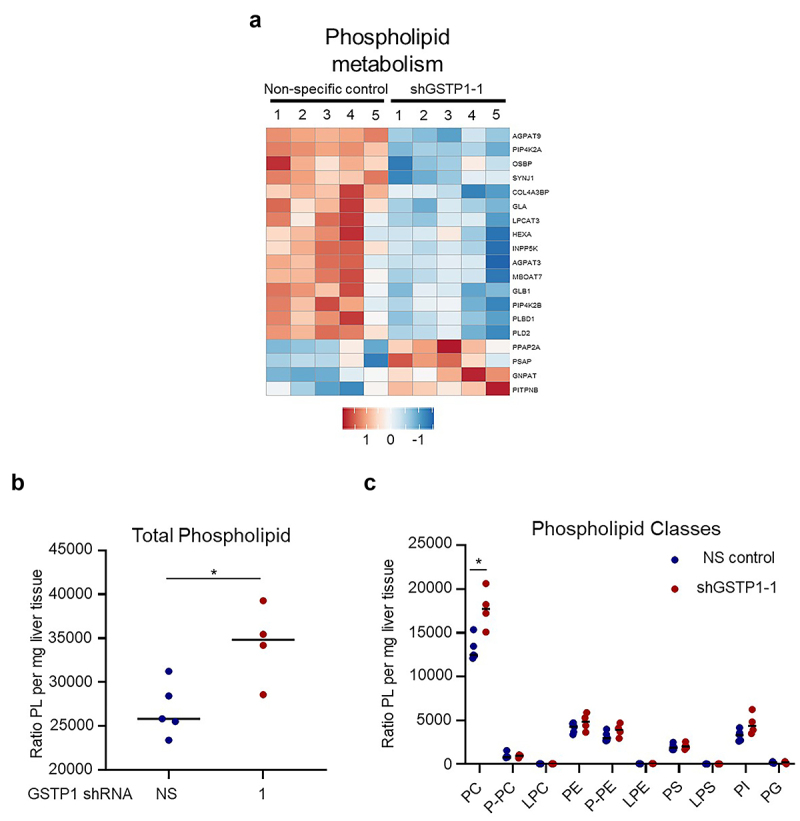
GSTP1 knockdown alters phospholipid metabolism in PDAC cells. (a) Proteomic pathway analysis identifies differential expression of proteins involved in phospholipid metabolism following GSTP1 depletion. Blue indicates downregulation and red indicates upregulation. (b) Total phospholipid content significantly increases in GSTP1-knockdown cells, suggesting compensatory lipid remodeling. (c) Phosphatidylcholine (PC) levels are elevated in GSTP1 knockdown cells, potentially reflecting oxidative stress-driven lipid alterations. Data are presented as representative results from 5 independent samples of NS control and shGSTP1–1 MIA PaCa-2 cells (*n* = 5). Statistical significance was determined using the student’s t-test, with * indicating a significant difference (*p* < .05) between GSTP1 knockdown and control conditions. Abbreviations: phosphatidylcholine (PC), plasmenyl phosphatidylcholine (P-PC), lyso-phosphatidylcholine (LPC), phosphatidylethanolamine (PE), plasmenyl phosphatidylethanolamine (P-PE), lyso-phosphatidylethanolamine (LPE), phosphatidylserine (PS), lyso-phosphatidylserine (LPS), phosphatidylinositol (PI), and phosphatidylglycerol (PG).

Based on the extensive metabolic disruptions observed, we next examined whether GSTP1 loss influences lipid metabolism, a critical component of PDAC adaptation to metabolic stress. Proteomic pathway analysis revealed differential expression of proteins involved in phospholipid metabolism, highlighting GSTP1’s potential role in lipid biosynthesis, remodeling, and membrane homeostasis (Figure 6a). These proteins regulate key lipid processes, including phospholipid synthesis, lipid signaling, and membrane composition, suggesting GSTP1 plays an integral role in coordinating lipid regulatory networks. To further explore this connection, we conducted a targeted, comprehensive phospholipid panel analysis to profile individual lipid species, including their fatty acid composition and relative abundances. Interestingly, despite the overall suppression of lipid metabolism genes and proteins, GSTP1 knockdown led to a significant increase in total phospholipid content in GSTP1 knockdown cells compared to control cells (Figure 6b). Among the phospholipid classes examined, phosphatidylcholine (PC) levels were significantly elevated following GSTP1 loss (Figure 6c). This finding was unexpected, given the observed transcriptional and proteomic downregulation of lipid metabolism pathways. As a key structural and signaling lipid, PC plays an essential role in maintaining membrane integrity and modulating lipid-mediated cellular processes. These findings suggest that the observed phospholipid accumulation may result from compensatory lipid remodeling, disruptions in lipid turnover or trafficking, oxidative stress-induced lipid modifications, or mitochondrial dysfunction. While the precise mechanism remains unclear, these changes highlight a complex relationship between GSTP1 and lipid metabolism in PDAC.

## 4. Discussion

Our findings establish GSTP1 as a critical regulator of metabolic homeostasis in PDAC. GSTP1 knockdown led to widespread metabolic reprogramming, characterized by the downregulation of key metabolic regulators (ALDH7A1, CPT1A, SLC2A3, and PGM1), ATP depletion, and mitochondrial dysfunction. Given that metabolic reprogramming is a hallmark of cancer, supporting tumor survival and progression, our results indicate that GSTP1 plays a broader role in tumor metabolism beyond its canonical function in redox homeostasis and detoxification.^20,24,32,33^ These findings suggest that GSTP1 loss creates a bioenergetic bottleneck by simultaneously impairing glucose uptake, glycogen metabolism, and fatty acid oxidation, potentially sensitizing PDAC cells to metabolic stress-inducing therapies.

While our primary focus was on metabolic genes and proteins downregulated upon GSTP1 knockdown due to their clear roles in lipid and carbohydrate metabolism, we also identified a small number of metabolism-related genes that were upregulated at both the transcript and protein levels. These included UPP1 (uridine phosphorylase 1) and DDAH1 (dimethylarginine dimethylaminohydrolase 1). UPP1 is involved in nucleotide metabolism, and although DDAH1 has roles in nitric oxide signaling and oxidative stress regulation, neither gene directly contributes to lipid or carbohydrate metabolism, which were the central focus of this study. Notably, DDAH1 was functionally validated in our previous work,^24^ and its upregulation further supports the connection between GSTP1 loss and altered redox signaling. These upregulated targets did not demonstrate clustering within the glycolytic or lipid metabolism pathways enriched in our analysis, reinforcing our focus on downregulated regulators that define GSTP1’s role in metabolic reprogramming in PDAC.

One key mechanism linking GSTP1 depletion to metabolic dysregulation may be the activation of AMP-activated protein kinase (AMPK). GSTP1 knockdown has been associated with increased AMPK phosphorylation, which serves as a cellular energy sensor that responds to low ATP levels by downregulating anabolic processes and upregulating catabolic pathways.^23^ Increased AMPK activity suppresses mTOR signaling, which in turn can impair lipid metabolism and mitochondrial function.^34^ Notably, ALDH7A1 and CPT1A downregulation has been observed in response to AMPK activation in other cancers.^35,36^ Additionally, the suppression of SLC2A3 and PGM1, both involved in glucose metabolism, suggests that AMPK activation could be shifting PDAC cells toward an energy-conserving state, further exacerbating metabolic dysfunction. Given that AMPK plays a crucial role in maintaining metabolic homeostasis under stress conditions, its activation in GSTP1-knockdown cells may represent a compensatory response to ATP depletion rather than a direct consequence of oxidative stress. Future studies should examine its phosphorylation status and downstream signaling pathways in GSTP1-deficient PDAC cells to determine its precise role in mediating metabolic changes.

In addition to AMPK activation, GSTP1 interacts with key stress signaling pathways, including the mitogen-activated protein kinase (MAPK) pathway. Specifically, GSTP1 inhibits c-Jun N-terminal kinase (JNK).^20,37,38^ GSTP1 knockdown leads to JNK hyperactivation, increasing c-Jun phosphorylation and altering stress-responsive gene expression.^37,38^ Similarly, GSTP1 prevents the autophosphorylation of apoptosis signal-regulating kinase 1 (ASK1) through interaction with tumor necrosis factor (TNF)-receptor-associated factor 2 (TRAF2), suggesting that its loss could amplify oxidative stress and metabolic dysfunction.^39,40^ While the precise contributions of these pathways to metabolic reprogramming remain unclear, they likely contribute to the broader stress response induced by GSTP1 depletion.

The reversibility of these metabolic changes upon GSTP1 restoration provides further evidence of GSTP1’s direct regulatory role in PDAC metabolism. Notably, while mRNA recovery often exceeded baseline levels, protein restoration was more variable. This discrepancy indicates that post-transcriptional regulatory mechanisms, such as mRNA stability, translation efficiency, or protein degradation pathways, may influence protein abundance. For instance, despite elevated mRNA levels, miRNA activity, RNA-binding proteins, or stress-induced translational repression could limit protein synthesis. Additionally, rapid protein turnover via the ubiquitin-proteasome system or autophagy-mediated degradation may selectively regulate metabolic proteins, preventing their accumulation. Another possibility is that specific cofactors, chaperones, or post-translational modifications are required for protein stability, delaying full recovery. Understanding these regulatory layers will be essential for identifying therapeutic strategies targeting GSTP1-mediated metabolic vulnerabilities in PDAC.

The incomplete restoration of metabolic gene expression following NAC treatment suggests that oxidative stress is not the sole driver of GSTP1-dependent metabolic changes. One possible explanation is GSTP1’s interaction with nuclear factor erythroid 2-related factor 2 (NRF2), the master regulator of cellular antioxidant responses.^41,42^ In a constitutive GSTP1 knockdown PDAC cell model, NRF2 mRNA expression was downregulated, indicating a broader disruption in antioxidant signaling that extends beyond ROS scavenging.^43^ Given that KRAS and Myc can upregulate NRF2 transcription to promote tumorigenesis, the loss of GSTP1 may interfere with these adaptive stress responses, further impairing metabolic homeostasis.^42,44^ Additionally, pharmacological inhibition of GSTP1 has been reported to induce nuclear translocation of NRF2, which suggests that GSTP1 depletion may lead to either NRF2 suppression or dysregulated activation depending on the cellular context.^32^ Furthermore, NRF2 is known to interact with AMPK and mTOR signaling, both of which were implicated in the metabolic alterations observed in GSTP1-knockdown cells.^45^ The interplay between these pathways could contribute to cell-line dependent variations in metabolic recovery following antioxidant treatment. Future studies should evaluate NRF2 nuclear translocation and its downstream targets in GSTP1-knockdown cells to elucidate this relationship in PDAC further.

GSTP1 also plays a role in lipid peroxidation detoxification through its regulation of ALDH7A1, a key enzyme that neutralizes toxic lipid peroxidation byproducts like 4-HNE.^46,47^ Further, ALDH7A1 contributes to energy metabolism by catabolizing 4-HNE to 4-hydroxy-2-nonenoic acid (4-HNA) and NADH, ultimately synthesizing acetyl CoA for fatty acid oxidation.^35,48^ ALDH7A1 downregulation following GSTP1 knockdown led to increased 4-HNE accumulation, contributing to mitochondrial dysfunction and oxidative stress. The complete restoration of ALDH7A1 expression with NAC treatment suggests that its regulation is tightly linked to oxidative stress. This aligns with previous studies showing ALDH7A1 upregulation as a protective response to oxidative damage in PDAC.^49^

Interestingly, ALDH7A1 has recently been identified as the most abundant ALDH isoform in PDAC, where it is associated with tumorigenicity and poor prognosis.^48^ ALDH7A1 has been implicated in tumorigenicity across multiple cancers,^50–52^ with roles in cancer-stem cell characteristics,^53^ metastasis,^54^ and cell cycle regulation.^47^ Given its role in energy metabolism and fatty acid oxidation, future studies should investigate whether targeting ALDH7A1 could further sensitize GSTP1-deficient PDAC cells to metabolic stress. However, while GSTP1 is known to contribute to redox homeostasis, its role in regulating lipid peroxidation pathways through ALDH7A1 has not been explored. Recent findings have shown ALDH7A1 expression in PDAC can additionally be regulated post-transcription through interaction with epidermal growth factor receptor kinase substrate 8 (EPS8), inhibiting proteasomal degradation,^55^ and via DNA-methylation in lung squamous cell carcinoma.^56^

In addition to lipid peroxidation detoxification, GSTP1 knockdown disrupted fatty acid oxidation through CPT1A suppression. CPT1A, the rate-limiting enzyme for mitochondrial fatty acid transport, is critical for ATP production in PDAC. Its downregulation upon GSTP1 loss suggests a metabolic shift that increases reliance on alternative metabolic pathways. CPT1A has been shown to promote the proliferation, migration, invasion, and chemoresistance of multiple cancers.^57–61^ CPT1A is also frequently overexpressed in PDAC and has been associated with PDAC progression and poor prognosis.^62,63^ While NAC treatment partially restored CPT1A expression, the incomplete recovery suggests that additional regulatory mechanisms, such as post-translational modifications, may influence its stability. CPT1A has also been found to have functions beyond fatty acid oxidation, as it has been implicated in antioxidant capacity and ferroptosis resistance in cancer cells.^64,65^ Given that ferroptosis is an iron-dependent form of cell death triggered by lipid peroxidation, CPT1A may contribute to redox homeostasis in a manner that is not fully restored by NAC alone.

Furthermore, GSTP1 is known to mediate S-glutathionylation, a post-translational modification that regulates protein function and stability.^21^ The absence of GSTP1-mediated glutathionylation in knockdown cells may contribute to CPT1A protein instability or altered enzymatic activity, further limiting its recovery. This supports our idea that GSTP1 may regulate CPT1A expression through mechanisms not solely related to lipid metabolism, as well as explain the partial recovery of CPT1A as cells attempt to compensate for metabolic disruptions caused by GSTP1 knockdown. CPT1 knockdown has been found to significantly reduce cell proliferation and alter mitochondrial morphology independently of fatty acid oxidation.^64^ While our findings demonstrate a strong link between GSTP1 function and the maintenance of optimal lipid balance in PDAC cells, it is essential to conduct further research to comprehend this relationship fully.

Beyond lipid metabolism, GSTP1 knockdown impaired glucose metabolism, as evidenced by SLC2A3 and PGM1 downregulation. Our data is supported by the previous finding that GSTP1 modulates glycolysis in triple-negative breast cancer cells.^23^ SLC2A3, a high-affinity glucose transporter, is frequently upregulated in cancers to support glycolysis.^66–70^ Our research demonstrates, for the first time, that GSTP1 regulates both the mRNA and protein expression of SLC2A3. The reduced expression of SLC2A3 upon GSTP1 knockdown suggests restricted glucose availability that may further compromise glycolytic flux and energy production. Interestingly, while SLC2A3 mRNA levels fully recovered with NAC treatment, protein restoration was variable, suggesting additional post-transcriptional regulation may limit its translation or stability.

One potential mechanism involves HIF-1α, a key regulator of glycolysis that is influenced by oxidative stress.^71^ GSTP1 knockdown disrupts redox homeostasis, which can either stabilize or degrade HIF-1α, depending on the redox environment. In a hypoxic environment, increased ROS promotes HIF-1α stabilization, enhancing the transcription of SLC2A3 to compensate for reduced oxygen availability. However, excessive oxidative stress can also impair HIF-1α stability and transcriptional activity, potentially disrupting SLC2A3 expression.^71^ Future studies are needed to determine whether HIF-1α plays a role in GSTP1-mediated metabolic regulation.

PGM1, which is involved in glycogen metabolism and glycolysis, was also significantly downregulated, potentially limiting glucose utilization and energy production.^72^ Given its emerging role as a metabolic vulnerability in various cancers, its regulation by GSTP1 suggests a previously unrecognized link between redox balance, GSTP1, and carbohydrate metabolism in PDAC.^73–76^ GSTP1 has been previously associated with the pentose phosphate pathway by interacting with lactic acid and leading to increased tumorigenesis, providing yet another point of evidence of GSTP1’s role in metabolic reprogramming to support cancer cell growth.^77^ The failure of PGM1 to recover with NAC treatment suggests that its suppression may be driven by additional factors beyond oxidative stress, such as transcriptional repression by NRF2, AMPK, or HIF-1α-mediated metabolic shifts. Further investigations into PGM1’s role in PDAC metabolism could reveal novel therapeutic opportunities.

Mitochondrial dysfunction and ATP depletion were among the most pronounced consequences of GSTP1 knockdown. Given GSTP1’s known role in redox homeostasis, increased oxidative stress likely contributed to mitochondrial impairment. In addition to its normal localization in the cytosol and nucleus, GSTP1 has also been located in the mitochondria of mammalian cell lines, raising the possibility that it may have direct mitochondrial functions that warrant further investigation.^78^ The varying degrees of mitochondrial dysfunction observed across PDAC cell lines may be linked to established metabolic subtypes, with glycolytic tumors exhibiting greater sensitivity to GSTP1 depletion. MIA PaCa-2 has been identified as a highly glycolytic subtype, relying heavily on aerobic glycolysis for ATP production.^11^ HPAF-II is characterized as a lipogenic PDAC subtype but has a higher glycolytic reliance than other lipogenic lines.^12^ Finally, PANC-1 is characterized as a lipogenic cell line but maintains a balanced energy production between lipid metabolism and glycolysis. MIA PaCa-2 and HPAF-II’s higher dependences on glycolysis may exhibit greater dysfunction upon GSTP1 loss due to impaired antioxidant defenses and redox homeostasis. Conversely, the more moderate mitochondrial dysfunction observed in PANC-1 suggests that its metabolic flexibility may allow for partial compensation when GSTP1 is lost.

Additional studies have found similar high and low-risk metabolic subtypes by analyzing the expression patterns of prognostic metabolic genes.^79,80^ The decrease in ATP observed in our study suggests that GSTP1 knockdown disrupts these adaptive mechanisms, potentially making PDAC cells more vulnerable to metabolic stress. GSTP1’s regulatory functions extend beyond detoxification, interacting with cellular signaling and stress responses, which more than likely have an additional impact on mitochondrial health and cellular energy status.^20,22,81^ Future studies should explore whether GSTP1 interacts with oxidative phosphorylation regulators or mitochondrial stress response pathways.

In support of our metabolite and ATP measurements, pathway-level analysis revealed broader disruptions across central metabolic networks in GSTP1-deficient PDAC cells. Both enrichment-based (MSEA) and topology-informed analyses identified significant alterations in pathways involved in amino acid metabolism, nucleotide turnover, and redox-related processes. These included key routes such as glutamate and cysteine metabolism, which are closely tied to glutathione synthesis and oxidative stress regulation. In addition, purine metabolism and other biosynthetic pathways required for energy and nucleotide production were affected. The convergence of these results with our transcriptomic and proteomic findings reinforces GSTP1’s role as a central coordinator of metabolic resilience. Its loss leads to a collapse in both energy-generating and protective pathways, rendering PDAC cells more vulnerable to oxidative and bioenergetic stress. These systems-level disruptions highlight potential metabolic vulnerabilities that could be therapeutically exploited in GSTP1-deficient tumors.

Despite the overall suppression of lipid metabolism pathways, GSTP1 knockdown led to a significant increase in phosphatidylcholine (PC) levels, suggesting a compensatory lipid remodeling response. PC is a major component of cellular membranes and plays a role in lipid signaling, membrane dynamics, and tumor progression.^82^ Lipid mediators derived from PC play an important role in modulating interactions between cancer cells and immune cells, influencing tumor progression and immune responses.^83^ This perspective could provide a broader context for understanding the implications of altered PC metabolism in PDAC. The observed increase in PC may reflect alterations in lipid synthesis, impaired degradation, or oxidative stress-induced lipid modifications.^82^ While the exact mechanism remains unclear, these findings suggest that GSTP1 is involved in maintaining lipid metabolism, potentially through interactions with lipid metabolism regulators.^23,84^ Further research is needed to determine whether these changes contribute to PDAC progression or represent a metabolic vulnerability that could be exploited therapeutically.

### 4.1. Implications for PDAC therapy and future directions

Given that elevated GSTP1 expression is associated with poor survival in PDAC patients, targeting GSTP1-mediated metabolic pathways may provide a novel approach to improve clinical outcomes. In addition to genetic knockdown, pharmacologic inhibition of GSTP1 represents a promising therapeutic strategy. Several classes of GSTP1 inhibitors have been identified, including covalent G-site inhibitors, H-site binders, and natural compounds such as piperlongumine, which has demonstrated anti-tumor activity in PDAC xenograft models.^21^ These agents inhibit GSTP1 enzymatic activity, disrupt its protein-protein interactions, and promote oxidative stress, thereby enhancing sensitivity to chemotherapeutics. Given our findings that GSTP1 depletion induces metabolic stress and impairs redox balance, future studies should evaluate the efficacy of these inhibitors, alone or in combination with metabolic stress-inducing agents, in PDAC models to assess their therapeutic potential.

The suppression of CPT1A and SLC2A3 suggests that inhibitors targeting fatty acid oxidation (e.g., etomoxir) or glycolysis (e.g., WZB117) may be particularly effective in GSTP1-deficient tumors. Additionally, the accumulation of 4-HNE suggests that combining GSTP1 inhibition with oxidative stress-inducing therapies, such as glutathione depletion or ferroptosis inducers, could further sensitize PDAC cells to metabolic stress.^84,85^ Further studies should evaluate the therapeutic potential of these combination strategies in preclinical PDAC models.

While this study establishes GSTP1 as a key regulator of PDAC metabolism, several questions remain. Future research should explore how GSTP1 influences metabolic gene regulation at the transcriptional and post-translational levels. Additionally, the in vivo relevance of these metabolic changes should be validated using patient-derived xenografts or genetically engineered mouse models. To more definitively establish a direct role for GSTP1 in regulating PDAC metabolism, future studies should also explore the effects of GSTP1 overexpression. Specifically, comparing the metabolic rescue capacity of wild-type versus catalytically inactive GSTP1 following doxycycline withdrawal would help determine whether the observed effects are dependent on GSTP1’s enzymatic activity. These studies would complement the current knockdown and recovery data and provide mechanistic clarity on whether GSTP1 contributes to metabolic regulation through its catalytic function or through protein-protein interactions and signaling regulation. Finally, understanding whether GSTP1 depletion universally disrupts metabolism across all PDAC subtypes will be critical for identifying patients most likely to benefit from GSTP1-targeted therapies.

This study highlights the essential role of GSTP1 in maintaining metabolic homeostasis in PDAC, bridging redox balance with lipid and glucose metabolism. Depletion of GSTP1 disrupts key metabolic pathways, resulting in ATP depletion, mitochondrial dysfunction, and the accumulation of lipid peroxidation products. By addressing this critical gap in knowledge, our study lays the groundwork for future research into GSTP1-mediated metabolic adaptations and their contribution to PDAC progression. These results reveal potential metabolic vulnerabilities in GSTP1-deficient PDAC cells, which could be targeted for therapeutic benefit.

## Supplementary Material

Figure_S2.tif

Figure_S4.tif

Figure_S1.tif

Figure_S3.tif

Figure_S5.tif

## Data Availability

All data supporting the findings of this study are included within this manuscript and its supplementary material. Additional datasets or specific raw data supporting the conclusions of this study are available from the corresponding author upon reasonable request.
