# Supplementary figures and images for: GSTP1 knockdown induces metabolic changes affecting energy production and lipid balance in pancreatic cancer cells
Source: Mol Cell Oncol. 2025 Jun 14;12(1):2518773. doi: 10.1080/23723556.2025.2518773 (PMC12169041; doi:10.1080/23723556.2025.2518773)

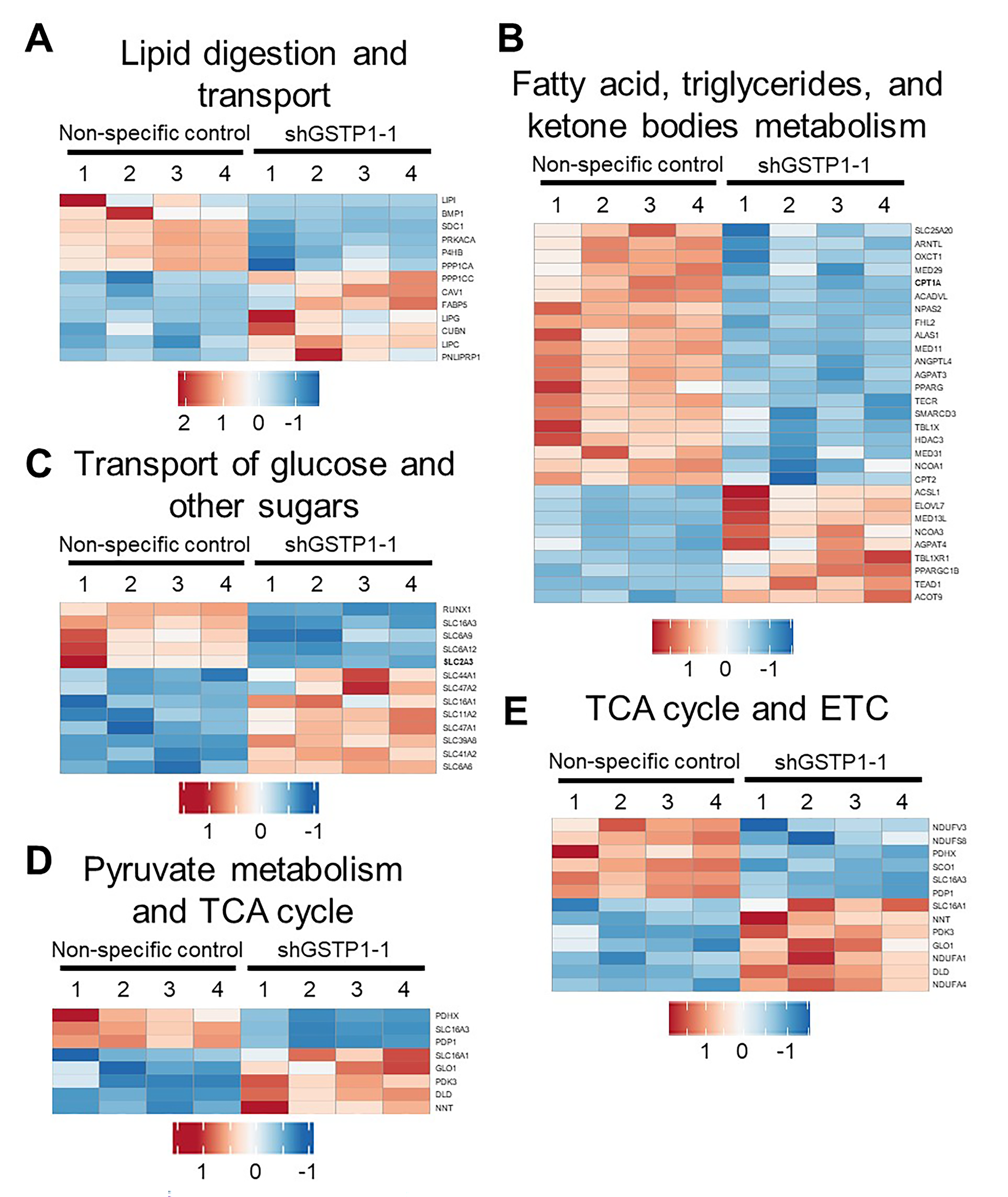

Supplement: Figure_S1.tif [file KMCO_A_2518773_SM8057.tif]

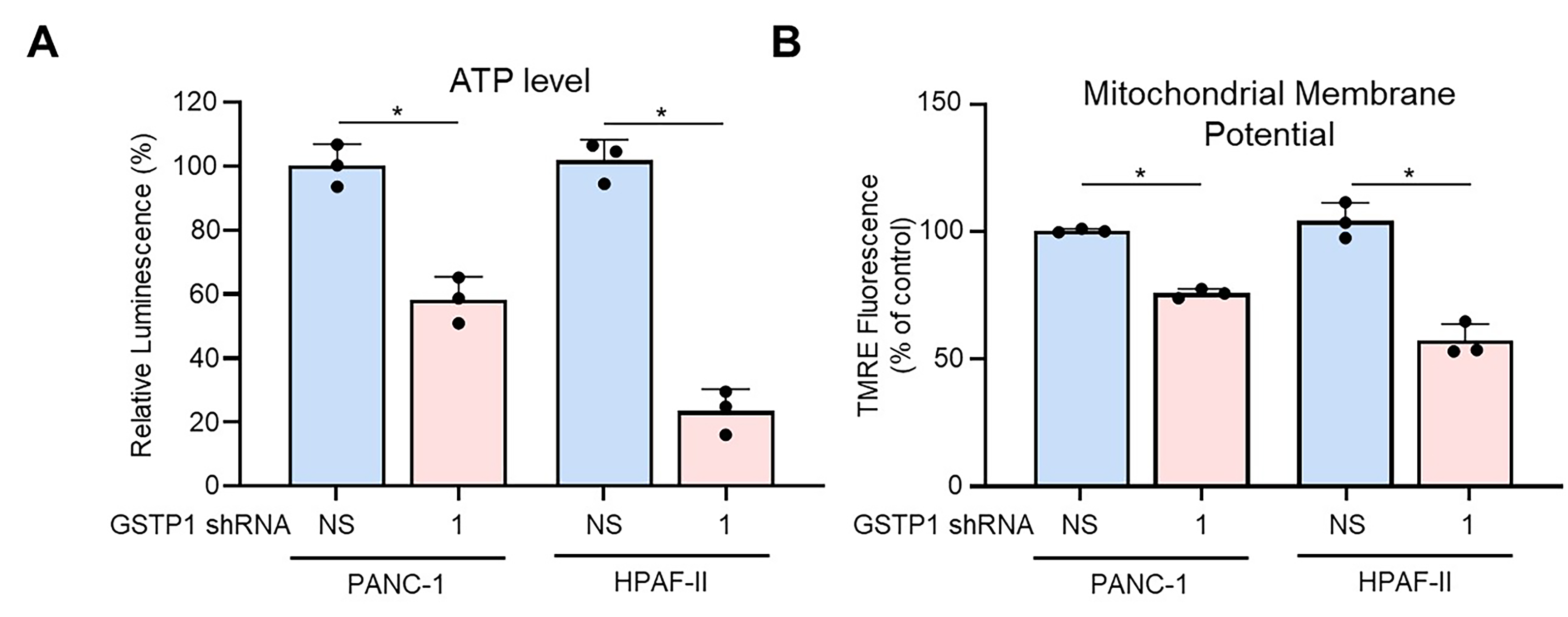

Supplement: Figure_S5.tif [file KMCO_A_2518773_SM8055.tif]
